# Six Letter with Replies

**Published:** 1879-04

**Authors:** 


### Our Correspondence.

PHILADELPHIA, March 1, 1879.

DEAR DOCTOR:

In my readings, I ran across the enclosed article the other day, which bears upon your heory of the contamination of well water adjacent to Cemeteries.

J. B. C.

"PROXIMITY OF WELLS TO CEMETRIES.—Dr. J. Lepert in a paper on the contamination of wells, by the washings of burying grounds, states that the analysis of the water of wells, contiguous to places of interment frequently brings to light a high degree of vitiation—the substances present, from their nitrogenous character, being clearly traceable to the adjoining burial place. In the case of a very deep well, more than three hundred feet from the nearest graves, the water, though bright and clear, was found to contain a very large proportion of ammoniacal salts, and on evaporation yielded an extraordinary quantity of dark colored organic matter mixed with carbonated salts. On the addition of hydrochloric acid to this residue, an offensive carbonic acid gas was given off, the smell being somewhat akin to that afforded by a mixture of a concentrated solution of glue and butyric acid. In summer, this water has a rancid taste, and when taken from the well, quickly becomes putrid. The author concludes that in any soil three hundred feet is not far enough away, whatever the depth of the well, and that in many soils it is practically useless in preventing the contamination of the well water; twice this distance if it be through porous strata, not sufficing to cut off the access of organic matter."

---

URBANA, OHIO, Feb. 20, 1879.

DR. UP DE GRAFF:

*Dear Sir:*—I was much impressed with your article upon Diphtheria, in the last BISTOURY. It throws light upon the cause of the epidemic which we have experienced in this place. Our Cemetery is upon a gravelly knoll. Near it is a street running parallel with the foot of the Cemetery. Here, occurred the first cases of the disease in our town, almost all of which proved fatal, and from which the other cases originated. The wells must necessarily receive water from the Cemetery, as they are in the natural drainage course from that mound to the creek, not far away.

It is well that the people should understand the importance of wholesome water, and I am glad the BISTOURY is teaching them the danger of drinking from wells located near Cemeteries. Yours truly, J. R. D.

We have received fourteen letters from as many different parts of the United States, all relating cases of Diphtheria, that occurred at the foot of Cemeteries. But we have published a sufficient number, we think, to call the attention of our readers to the danger of using wells in such localities.

---

PHILADELPHIA, Jan. 23, 1879.

THAD. S. UP DE GRAFF, M. D.:

*Dear Sir:*—A copy of the "BISTOURY" sometimes gets into my hand; and, after reading it, I feel as if your surgical opinion would be as valuable to me, as that of any one.

I am twenty-four years old. Fourteen years ago, I fell and broke my nose, producing considerable flattening of nasal bones and cartilages. Can anything be done to remove the deformity? E. B.

Better consult some surgeon in your own city. We cannot give an opinion, without seeing the extent of the injury.

---

BLACKSTONE, MASS., Jan. 17, 1879.

DEAR DOCTOR:

Can you send me unbound numbers of the BISTOURY, four or five years old? I would like those of the years 1872–3.

W. R. MCLAREN, M. D.

Yes, price Fifty Cents a year, and four cents for postage. We can send you bound volumes as follows: 1869 to 1872; 1872 to 1875; 1875 to 1878, at $2.25 for each bound volume.

---

CANTON, PA., Feb. 25, 1879.

DR. UP DE GRAFF:

*Dear Sir:*—In support of the theory that the existence and prevalence of diphtheria depends upon impure water and foul air, I cite the following cases that occurred in my practice in 1878.

*Case 1st.*—A family consisting of the parents, four children and servant girl, living in a house below and near the old Canton Cemetery, were attacked in January. Three of the children had true diphtheria. The father had diphtheroidal sore throat.

The rest of the family had diphtheritic sore throat. About a year previous to this attack, the mother had a long, severe run of typhoid fever, and rarely a month passed, for years, but some of the family required the attendance of a physician. Previous to the spring of 1878, they used water from a well which was located near the house.

It is now nearly a year since they have been supplied with water, by the "Canton Water Company," and in that time they have all enjoyed excellent health.

*Case 2nd.*—A family of five; parents and three children, living five miles east of Canton, attacked in May, two of the family had true diphtheria. The rest of the family had diphtheritic sore throat.

A son eighteen years old, was sick nine weeks, the disease refusing to yield to treatment, until the patient was removed to another locality. A niece of the father, with her husband, went to assist in nursing the sick. They contracted the disease, and were sick for four weeks, without showing any signs of improvement. They were then removed a distance of five miles to their own residence, where they recovered rapidly. Well located in the chip-yard, surrounded by decaying and rotten wood. Cellar under their home always damp.

*Case 3rd.*—Family of six; parents and four children, residing twelve miles west of Canton, in Tioga County, attacked in July. Three daughters had true diphtheria. The father and son had diphtheritic sore throat. The mother had diphtheroidal sore throat.

House and barn located on a side-hill, privy above the house, and without vault, used water from a shallow well, which was sunk in a swampy piece of ground, six to eight rods below the buildings.

*Case 4th.*—Family residing on the line of the N. C. R. R., ten miles southwest of Canton, attacked in September, two of the family had true diphtheria, the others had sore throat. Used water from a well, which was sunk in a gravelly, porous soil. In wet weather, the well fills up and overflows, but in dry weather, the water sinks to the level with a creek that flows near the house.

There are a number of privy vaults, within five to ten rods of the well.

*Case 5th.*—A family of four; parents and two children, living twelve miles south of Canton, in Sullivan County. Children had true diphtheria. House and privy without vault, located on side-hill. Swampy land below the house, from which issues a spring that supplies the family with water.

*Case 6th.*—A family consisting of the parents, four children and two hired help, attacked in November. Three of the children had true diphtheria, all the rest of the family were sick with sore throat. Used well water, but the well was favorably located. Near the back kitchen door, was a large cess pool where all the slops from the house were thrown, and from which emanated a nauseating stench.

I mention these cases, as they were the first that occurred in the several localities mentioned, and originated in families that had no communication with those that had contracted the disease. The disease was communicated in every case cited, to others in the neighborhood, by direct contact with the sick.

J. E. C., M. D.

---

LEBANON, O., March 4, 1879.

DR. THAD S. UP DE GRAFF:

*Sir:*—I am recommended to write you concerning my eyes. I had an operation performed on one of them for cataract, about nine years ago, and it proved a failure, and in consequence, the sight of my other eye, is almost entirely destroyed. But yet I can see to count and distinguish colors. And with my eyes either shut or open, I can see all manner of sights imaginable, and among the rest, an object which seems to be the size of an acorn, with a tail to it about two feet long. Sometime I seem to see boiling springs, and when I get my mind worked up, or get excited, there seems to be a running river before my eyes. At other times there seems to be something resembling a bale of cotton, all picked to pieces, or worm eaten.

When I am still, and don't move around much, my eyes are a great deal better, but when I exercise, and try to do anything, I see all these sights, and am almost totally blind.

I send you these statements of the case, so that if possible, you can tell me what is the matter. Write, and if you can explain my case, and tell me, if possible, what can be done. Please answer soon. Yours respectfully,

J. P. H.

The nerve or retina, or both, have been injured by sympathetic inflammation. Consult some good oculist.

